# Parent-offspring resemblance for educational attainment reduces with increased social class in a global sample: evidence for the compensatory advantage hypothesis

**DOI:** 10.3389/fpsyg.2023.1289109

**Published:** 2024-01-03

**Authors:** Michael A. Woodley of Menie, Matthew A. Sarraf, Mateo Peñaherrera-Aguirre, Heiner Rindermann

**Affiliations:** ^1^Independent Researcher, London, United Kingdom; ^2^Independent Researcher, Boston, MA, United States; ^3^Department of Psychology, College of Science, University of Arizona, Tucson, AZ, United States; ^4^Department of Psychology, Chemnitz University of Technology, Chemnitz, Germany

**Keywords:** compensatory advantage hypothesis, education, genetics, intelligence, Scarr-Rowe effect

## Abstract

**Introduction:**

The degree to which (self-reported) social class predicts parent-offspring resemblance for educational attainment (EA) is examined in a globally representative dataset of 69,116 individuals sourced from 56 countries.

**Methods:**

A hierarchical general linear model is used to predict participant EA with the two-way interaction between class and parental EA, after controlling for regional effects, the main effects of age, class, parental EA, and interactions among these.

**Results:**

Social class-by-parental EA interaction *negatively* predicts participant EA (*semipartial r* = −0.04, 95% CI = −0.05 to −0.03), meaning that among those who report belonging to a “higher” social class, the degree of parent-offspring resemblance for EA is reduced, contrary to the Scarr-Rowe hypothesis, which holds that genetic influences on cognitive ability and related phenotypes (captured here in part by parent-offspring resemblance) should be greater among those from higher socioeconomic status (SES) backgrounds. These results replicate using a quantile regression model, where it was found that among those with lower social class ordinals, the strength of the parent-participant EA association is significantly stronger relative to those in the highest ordinal. No significant sex differences are present.

**Discussion:**

These findings are consistent with the compensatory advantage hypothesis, which predicts decreased heritability of EA and related phenotypes among affluent families, as increased access to educational resources should enhance opportunities for cognitive growth in a way that compensates for intrinsic disadvantages.

## 1 Introduction

The Scarr-Rowe effect (also “interaction” or “hypothesis”) is a gene-by-environment interaction characterized by a reduction in the additive genetic variance of cognitive ability among those with relatively low socioeconomic status (Tucker-Drob and Bates, 2015), often thought to be due to the adverse developmental effects associated with such status. The existence of this effect was first theorized in the 1960s (Jensen, 1968), with Sandra Scarr (1936–2021) initially providing evidence for it in the 1970s (Scarr-Salapatek, 1971). Scarr argued on the basis of her findings that exposure to poverty, which is associated with restricted access to beneficial environmental influences on development, might be an important determinant of between-social-class and between-socially-identified-racial-group mean differences in cognitive performance within the US, insofar as variance in developmentally relevant environmental exposures across such social classes and groups would alter the heritability of cognitive ability across them (Scarr, 1981). David Rowe (1949–2003) led a team of researchers that replicated the effect 28 years later using data from the US Add Health cohort (Rowe et al., 1999). Subsequent researchers have used the term Scarr-Rowe “effect” and related terms (such as “interaction” or “hypothesis”) (e.g., Turkheimer et al., 2009; Woodley of Menie et al., 2018) in recognition of the pivotal role that these two behavior geneticists played in gathering evidence for the existence of the phenomenon.

Since the publication of Rowe et al. (1999), numerous studies have been published examining the effect in a number of different countries employing twin-study-based estimates of the heritability of cognitive ability. The picture that has emerged is complex, however. The meta-analysis of Tucker-Drob and Bates (2015) found, via re-analysis of previously published data, that statistically significant Scarr-Rowe effects (operationalized as SES-by-additivity interactions) were only present in the US. In their European sub-sample, the effect was negatively signed and non-significant. A subsequent study by Figlio et al. (2017) found that the effect is absent in a very large sample of Floridian twins and siblings representing more recent birth cohorts (those born in the 1990s and 2000s), although it should be noted that these researchers were not able to directly assign zygosity in their twin subsample. An Australian twin study found no evidence for the effect (Bates et al., 2016), and a more recent study employing Nigerian twins similarly failed to find evidence for the effect (Hur and Bates, 2019).

Other studies of the Scarr-Rowe effect have used different behavior-genetic techniques, including adoption designs (Loehlin et al., 2022), parent-offspring resemblance measures (Nagoshi and Johnson, 2005; Flores-Mendoza et al., 2017), and molecular methods such as single nucleotide polymorphism heritabilities and polygenic scoring (PGS) (Woodley of Menie et al., 2018; Rask-Andersen et al., 2021; Woodley of Menie et al., 2021; Peñaherrera-Aguirre et al., 2022). The results of these studies have also been mixed, with some (e.g., Woodley of Menie et al., 2018, 2021; Peñaherrera-Aguirre et al., 2022) finding evidence for the effect in both younger and older US cohorts, some finding evidence for the effect in (younger) Brazilian cohorts (Flores-Mendoza et al., 2017), some finding no evidence for the effect in (older) US cohorts (Nagoshi and Johnson, 2005; Loehlin et al., 2022), and another finding evidence for the opposite effect in a large middle-aged UK cohort (Rask-Andersen et al., 2021).

A number of studies have also investigated the Scarr-Rowe effect in relation to educational attainment (EA), and associated scholastic achievement criteria, utilizing both molecular-genetic and classic behavior-genetic approaches [see the review in Ruks (2022)]. Parental education (which includes formal qualifications as well as practiced education in families) is a good predictor of children’s development, both cognitive and academic. The typical effect size (*r/β*) is about 0.45 (Rindermann and Baumeister, 2015; Rindermann and Ceci, 2018). EA has been found to share approximately 60% of its genetic variance in common with direct measures of cognitive ability in twin studies (Johnson et al., 2006). PGSs estimated with respect to EA also predict variation in direct measures of cognitive ability (Okbay et al., 2016, 2022; Lee et al., 2018).

As with the Scarr-Rowe effect on cognitive ability, considerable heterogeneity with respect to the effect in relation to EA and associated measures has been noted, with some studies finding signs of negative moderation (i.e., apparent anti-Scarr-Rowe effects) on at least some measures of EA (e.g., Rask-Andersen et al., 2021; Ruks, 2022).

Heterogeneity among Scarr-Rowe effects with respect to time and region may stem from a variety of factors. For example, unusually robust institutions designed to promote greater equality and inter-generational mobility may explain the absence of the effect in certain European nations, as well as Australia, compared to the US. Highly generalized extreme poverty is reasonably expected to broadly limit life opportunities across social strata, possibly accounting for the absence of the effect in Nigeria (see Tucker-Drob and Bates, 2015; Hur and Bates, 2019; Baier et al., 2022 for further discussion). Differences in factors such as behavior-genetic measurement model, statistical power, outcome variable choice (e.g., narrow cognitive abilities, full-scale IQ, or educational attainment), and even the Wilson effect (i.e., the increase in heritability of IQ with age) might contribute to the notable variability in study results (see Tucker-Drob and Bates, 2015; Baier et al., 2022; Peñaherrera-Aguirre et al., 2022 for relevant discussion).

Anti-Scar-Rowe effects (e.g., Rask-Andersen et al., 2021) are more difficult for the classic Scarr-Rowe hypothesis to accommodate. Ruks (2022) proposed a recent behavior genetic extension of the sociological compensatory advantage hypothesis (CAH; Bernardi, 2014), which maintains that high-SES families compensate for intrinsic disadvantages of their members by provisioning environments that can cultivate cognitive growth and promote educational attainment. Such disadvantages may include a genetic propensity toward a lower level of EA. In so far as such families can overcome these disadvantages via compensatory provisioning of resources this may in some cases lead to an apparent of the Scarr-Rowe effect. This phenomenon is captured by the broader mechanism of compensation within the gene-by-environment interaction mechanism typology of Shanahan and Boardman (2009). Compensation effects arise when the influence of environmental enrichment on development is sufficiently strong as to prevent the realization of a phenotype to which “disadvantageous” genetic endowments predispose an individual (such endowments relevant to a given phenotype are sometimes collectively referred to as genetic diathesis [although this term is more often used in medical genetics]). Utilizing the German Twin Life sample, Ruks (2022) found evidence for the CAH on an EA measure (tertiary enrollment). It was noted that the effect was primarily associated with the moderating effect of SES on genes for cognitive ability.

In the current analysis a globally representative and very large sample of individuals sourced from the latest data release of the World Values Survey is used to test for the presence of both the Scarr-Rowe effect and the CAH at the cross-national level. Despite lacking twin data, the WVS nevertheless contains internationally harmonized data on both parental and offspring (participant) EA, and also participant (self-reported) social class. These data can therefore be used to detect a moderating effect of class on the degree of parent-offspring resemblance for EA. Parent-offspring resemblance cannot differentiate between genetic and environmental influences on cognitive ability (Devlin et al., 1997). But, as already noted, parent-offspring resemblance has been used in previous studies of the Scarr-Rowe effect in the absence of twin or other types of familial data that would permit the direct estimation of behavior-genetic variance components (e.g., Nagoshi and Johnson, 2005; Flores-Mendoza et al., 2017). A finding of moderation involving a simple parent-offspring resemblance model must therefore be interpreted with caution, as moderation stemming from SES on additive heritability is only one pathway that can influence the degree of parent-offspring resemblance.

## 2 Method

### 2.1 Data

All data are sourced from the newly released seventh wave of the World Values Survey (Haerpfer et al., 2022). This is a large-scale longitudinal data collection effort that started in 1981. For the current wave, data collection started in mid-2017, and was finalized by the end of 2021. Sampling is furthermore structured so as to maximize representativeness with respect to country-level demographics. The survey items are also designed to be maximally comparable in cross-cultural analysis. The WVS makes all data, including individual-level response data, freely available to those wishing to conduct their own analyses.^1^ After exclusion of participants with missing data for at least one variable, the final dataset included 69,116 individuals, covering 56 countries. The variables selected for use in this analysis are described below.

#### 2.1.1 Age

Participant age varied widely in these data, ranging from 16 to 103 years (*M* = 42.85 years, *SD* = 16.36 years). Age and birth year were established using two questions, the first asked participants, “Can you tell me your year of birth, please?” (Q261). This was followed by: “This means you are____ years old (*write age in two digits*)” (Q262, italics in original). Age (in years) was used to control for different levels of exposure to education, with younger participants likely having benefited more from greater access to education (due to modernization) than older ones in most regions (Barro and Lee, 2013).

#### 2.1.2 Educational attainment (EA)

EA was available for participants (Q275), their mothers (Q277), and fathers (Q278). This variable uses the International Standard Classification for Education employed by UNESCO and the UN, and corresponds to a nine-point scale encompassing the following categories: 0 = early childhood education, 1 = primary education, 2 = lower secondary, 3 = upper secondary, 4 = post-secondary non-tertiary, 5 = short-cycle tertiary education, 6 = bachelor degree or equivalent, 7 = master degree or equivalent, and 8 = doctoral degree or equivalent. Parental EA was averaged and used as a predictor. Participants for whom data on only one parent were available were excluded from the analysis. Parental correlation with respect to EA is very high in these data (*r* for mother’s EA predicting father’s EA = 0.778, *n* = 69,116 individuals). In contrast, the correlation between parental EA and Social Class was small in magnitude (*r* = 0.061, *p* < 0.0001, *n* = 69, 116 individuals).

#### 2.1.3 Social class

Social class includes vertically transmitted cultural legacy effects (e.g., socially inherited position) which go beyond SES (which usually captures only wealth and/or income and parental EA) (Deutsch, 2017). As the environments believed to be most relevant to the Scarr-Rowe effect are associated with childhood, rather than adulthood, and as direct measures of childhood social deprivation are not included in the WVS, social class (which is measured by the WVS) is used as a cultural proxy for these environments instead. It should also be noted that parental EA, which is sometimes used in studies of the Scarr-Rowe effect as a narrow index of childhood SES (e.g., Woodley of Menie et al., 2021), is employed here as the basis for estimating parent-offspring resemblance, and so cannot be used as a distinct moderator. The WVS asks about participant social class as follows: “[p]eople sometimes describe themselves as belonging to the working class, the middle class, or the upper or lower class. Would you describe yourself as belonging to the …” this is then followed by the administration of a five-point scale organized as follows: 1 = upper class, 2 = upper middle class, 3 = lower middle class, 4 = working class, and 5 = lower class (Q287). For the purposes of the current analysis, the class variable was recoded, so that self-reporting membership in a “higher” class category was associated with a higher ordinal. Participant’s self-reported social class aggregated at the country level positively correlates with cross-national indicators of prosperity (such as log-transformed GDP *per capita*, *r* = 0.486, 95%, CI = 0.256 to.664, *n* = 56 countries). This indicates that self-reported social class has transcultural validity in the WVS dataset, and that (at least in part) it is with respect to the global population that participants are making determinations concerning their relative social status. This is significant as local reference effects (e.g., when a participant makes a level judgment based largely on their national or sub-national context) can severely compromise the cross-cultural validity of more subjective measures (for discussion of this problem in relation to cross-national measures of personality, see Allik et al., 2012).

#### 2.1.4 Sex

Participant sex is coded as follows, 1 = male and 2 = female (Q260). This variable will be used to determine whether sex differences in the magnitude of any interactions between social class and parental EA predicting participant EA are present in these data.

#### 2.1.5 Biogeographic regional effects

It is possible that there are broad geographic influences on global variability in EA (e.g., Diamond, 1997; Rindermann, 2018). These factors may influence this variability above and beyond the effects of individual differences. In order to control for these regional effects, each individual is assigned to one of eight biogeographic regions based on their country of residence. This is similar to the approach used in the country-level studies of Figueredo et al. (2021a,b). The biogeographic regions include countries subsumed into the Neotropical, Afrotropical, Oriental, Nearctic, Palearctic, Oceanaian, and Sino-Japanese regions. These regions are then entered into the model as predictors. The correspondence between each biogeographic region and country is listed in Table 1.

**Table 1:** World Values Survey countries with their corresponding biogeographic regions.

Country	BGR	Country	BGR	Country	BGR
Andorra	PAL	Indonesia	ORI	Netherlands	PAL
Argentina	NEO	Iran	SAA	Pakistan	ORI
Armenia	PAL	Iraq	SAA	Peru	NEO
Australia	OCE	Jordan	SAA	Philippines	ORI
Bangladesh	ORI	Japan	SNJ	Puerto Rico	NEA
Bolivia	NEO	Kenya	AFR	South Korea	SNJ
Brazil	NEO	Kyrgyzstan	PAL	Romania	PAL
Canada	NEA	Kazakhstan	PAL	Russia	PAL
Chile	NEO	Lebanon	SAA	Singapore	ORI
China	SNJ	Libya	SAA	Serbia	PAL
Colombia	NEO	Maldives	ORI	Tajikistan	PAL
Cyprus	PAL	Malaysia	ORI	Taiwan	SNJ
Vietnam	ORI	Macau	SNJ	Thailand	ORI
Ecuador	NEO	Mexico	NEA	Tunisia	SAA
Egypt	SAA	Montenegro	PAL	Turkey	PAL
Ethiopia	AFR	Morocco	SAA	Ukraine	PAL
Germany	PAL	Myanmar	ORI	USA	NEA
Greece	PAL	New Zealand	OCE	Venezuela	NEO
Guatemala	NEO	Nicaragua	NEO	Zimbabwe	AFR
Hong Kong	SNJ	Nigeria	AFR		

BGR, Biogeographic region; NEO, Neotropical; AFR, Afrotropical; ORI, Oriental; NEA, Nearctic; PAL, Palearctic; OCE, Oceanaian; and SNJ, Sino-Japanese regions.

### 2.2 Measurement models

The current study computed a Hierarchical General Linear model (GLM) based on Type I Sum of Squares (SS1). In contrast to SS2, wherein the parameter estimates are calculated simultaneously, an SS1 GLM hierarchically partitions the model’s variance based on the order in which the predictors enter the system of equations. As the variance estimates associated with each interaction term are unique, the significance of each of these can be estimated without the need for multiple comparison correction (e.g., *α* = 0.05). Thus, the current study examined the influence of predictors based on the following order: (1) biogeographical region; (2) age; (3) parental EA; (4) social class; (5) the interaction between BGR and age; (6) the interaction between BGR and parental EA; (7) the interaction between BGR and social class; (8) the interaction between age and EA; (9) the interaction between age and social class; and (10) the interaction between parental EA and social class. For steps 1, 5, 6, and 7, set-level effects (squared multiple correlations) are presented (rather than the effects associated with each BGR separately). A Scarr-Rowe-like effect would manifest as a positively signed and statistically significant two-way interaction term, which would indicate that as participant social-class-level increases, so too does the degree to which parental average EA predicts participant EA. Conversely, an oppositely signed interaction (negative) would be consistent with predictions from the CAH, as parent-offspring resemblance for EA would be reduced among those reporting “higher” class ordinals.

GLM-type models that are saturated for both main and interaction effects theoretically account for larger portions of the variance in the dependent variable than those that specify only certain interactions, in addition to which they also permit confounding interactions to be thoroughly controlled, increasing confidence in the resultant outcomes of interest. Such saturated models have been used to successfully detect Scarr-Rowe and related effects in previous studies employing large sample sizes (Woodley of Menie et al., 2021; Peñaherrera-Aguirre et al., 2022). These data will also be broken out by participant sex in order to examine the presence of possible sex differences in effect magnitude. All analyses are conducted using *UniMult 2.0* (for documentation on the original version of *UniMult*, see Gorsuch, 1991).

As variable skewness can condition the outcomes of models testing for the presence of interaction terms (Martin, 2000), this parameter was estimated for all variables. The variables used all exhibited skewness values that fell between −/+2.00 (with most falling between −/+1.00), which is considered generally acceptable for psychometric purposes (George and Mallery, 2010). A quantile regression model was also conducted examining the effects of parental EA, social class, and the corresponding interactions on participant EA. This model was computed using the *quantreg* package (Koenker et al., 2018) in R v 4.0.1.

## 3 Results

**Figure 1:**
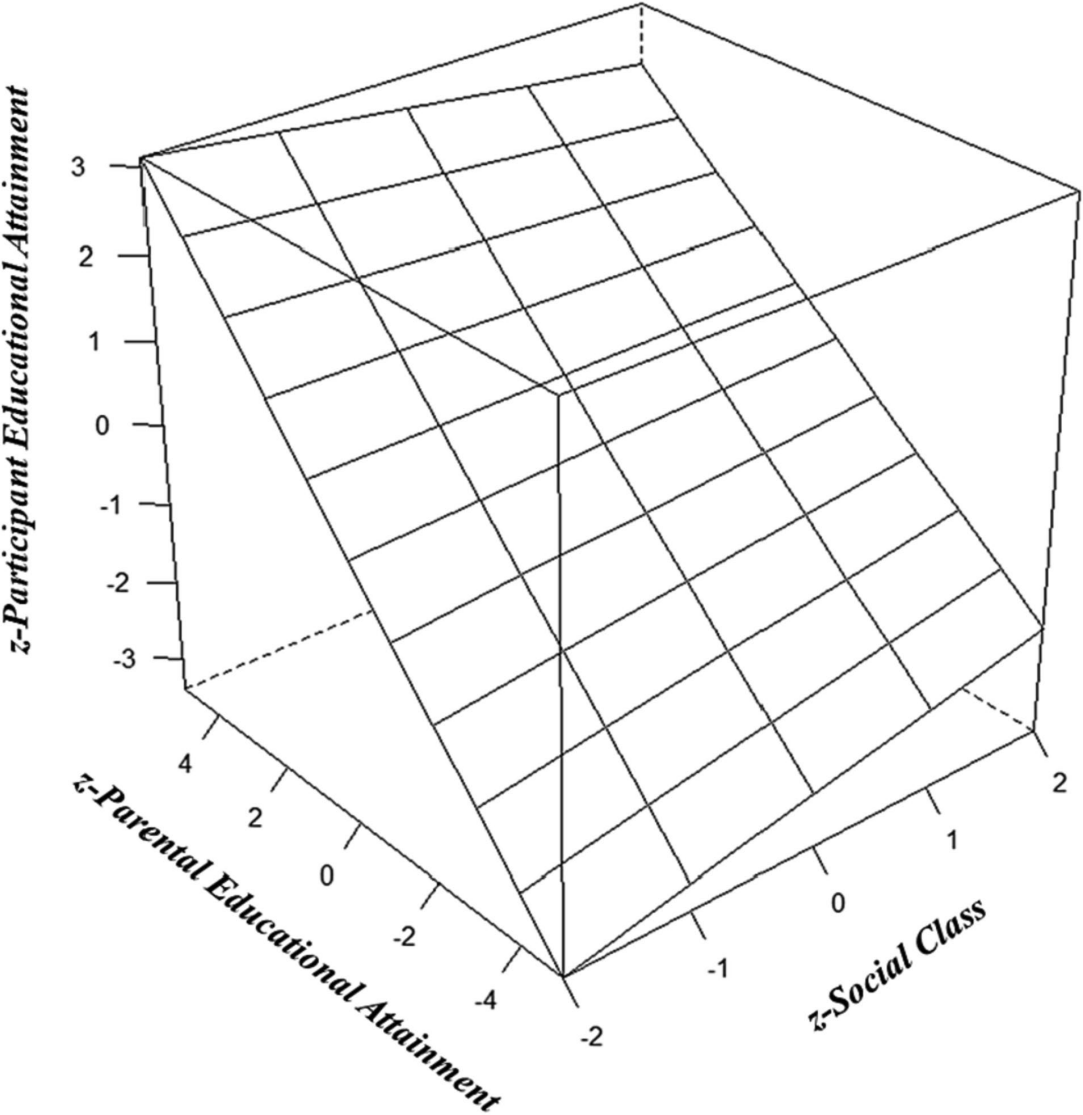
Regression plane plot visualizing the negatively signed interaction between parental educational attainment (EA) and social class on participant EA (consistent with the compensatory advantage hypothesis). This effect can be visualized by comparing the shallower slope of the association between parental and participant EA among those with higher social class to the steeper slope among those with lower social class.

As indicated in Table 2, the Hierarchical GLM multiple R reached statistical significance and explained 34.8% of the variance. The analysis revealed a positive and significant effect (these are scaled as *semi-partial regression coefficients* [*sr*], which can be interpreted analogously to standardized regression coefficients [*β*] in standard multilinear regression models) of the BGR set on participant EA. Age negatively and significantly predicted the criterion variable (*sr* = −0.18). Parental EA positively and significantly predicted participant EA (*sr* = 0.42), explaining a sizable proportion of the model’s variance. Social class was also a positive but small predictor of the criterion variable (*sr* = 0.07). The model furthermore revealed positive and significant interactions between biogeographic regions with age, parental EA, and social class. The interaction between age and parental EA did not predict participant EA. In contrast the age-by-social class interaction reached statistical significance. Consistent with the CAH, the model estimated a negative and significant interaction between parental EA and social class, indicating the presence of an anti-Scarr-Rowe effect (*sr* = −0.04). This interaction is illustrated with a regression plane plot (Figure 1). These correspond to the quantile weights used to estimate the regression coefficients.

**Table 2:** Hierarchical general linear model (SS1) examining the influence (expressed as a semi-partial regression coefficient [*sr*]) of BGR, age, parental EA, and social class on participant EA.

Predictors	Sr	95% CI	*F*-value	Df1/Df2	Value of *p*
Biogeographic region	0.35	0.34, 0.36	1856.87	7/69081	<0.0001
Age	−0.18	−0.19, −0.17	3257.93	1/69081	<0.0001
Parental EA	0.42	0.41, 0.43	18953.24	1/69081	<0.0001
Social class	0.07	0.06, 0.08	508.98	1/69081	<0.0001
Set 1					
Biogeographic region*Age	0.08	0.07, 0.09	98.26	7/69081	<0.0001
Set 2					
Biogeographic region*Parental EA	0.06	0.05, 0.07	52.62	7/69081	<0.0001
Set 3					
Biogeographic region*Social class	0.03	0.02, 0.04	15.61	7/69081	<0.0001
Age*Parental EA	0.00	−0.01, 0.01	0.00	1/69081	0.9000
Age*Social class	0.01	0.00, 0.02	8.26	1/69081	0.0040
Parental EA *Social class	−0.04	−0.05,− 0.03	140.97	1/69081	<0.0001
Multiple R	0.59	0.59, 0.59	1089.23	34/69081	<0.0001

The model also estimated the influence of the following interaction sets: region by age, region by EA, and region by social class, in addition to age by parental EA, age by social class, and finally, parental EA by social class.

**Figure 2:**
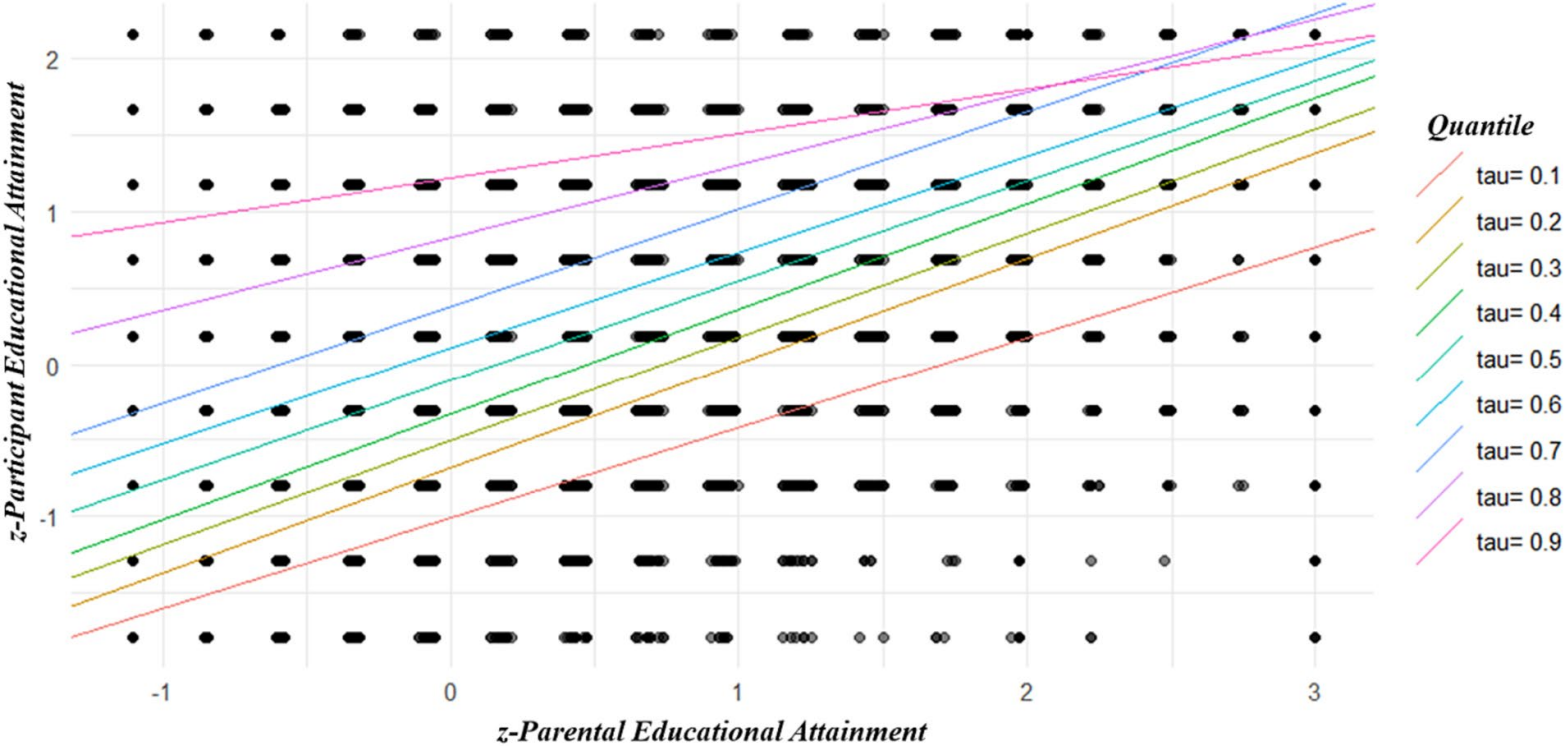
Quantile regression scatterplot illustrating the association between standardized parental EA and standardized participant EA as a function of various tau values.

A subset analysis was conducted to determine the magnitude of the interaction between parental EA and social class on the criterion variable for each sex separately. The hierarchical GLMs were specified following the same order as that described in Table 2. Both hierarchical GLMs revealed negative and significant interactions between parental EA and social class. As indicated in Table 3, however, no significant sex differences were detected when the outputs of the two models were compared. A quantile regression analysis revealed a positive and significant influence of parental EA on participant EA. It also found that the greater the social class the higher the participant EA (using the lowest social class ordinal as a baseline). Lastly, the model detected negative and significant interactions between parental EA with each social class ordinal on participant EA (using the lowest social class ordinal as a baseline). These results are further summarized in Table 4. A quantile regression scatterplot illustrates the slope variation as a function of various tau values (Figure 2).

**Table 3:** Fisher *r-z* significance test based on the semi-partial regression coefficients (*sr*) for the interaction between parental EA and social class predicting participant EA broken out by participant sex.

Sex	*sr*	95%CI		*F*-value	Df1/Df2	Value of *p*
Male	−0.03	−0.04, −0.02		55.01	1/33384	<0.0001
Female	−0.04	−0.05, −0.03		76.80	1/35662	<0.0001
Fisher *r*-*z* significance test						
*z*-value	Value of *p*					
1.32	0.094					

Main effects and other interactions are not shown.

**Table 4:** Quantile regression model examining the influence of parental EA, social class, and the corresponding interaction on participant EA.

Predictor	Estimate	Std. error	*t*-value	Value of *p*
Intercept	−0.408	0.010	−40.67	<0.00001
Parental EA	0.804	0.011	73.91	<0.00001
Social class (2)	0.317	0.010	31.32	<0.00001
Social class (3)	0.321	0.013	24.00	<0.00001
Parental EA * Social class (2)	−0.161	0.011	−14.47	<0.00001
Parental EA * Social class (3)	−0.163	0.012	−13.27	<0.00001

## 4 Discussion

A relatively small-magnitude negative effect of social class on parent-offspring resemblance for EA was identified, consistent with predictions from the compensatory advantage hypothesis (CAH). It should be noted that in psychological science the mean effect size [*r*] is approximately 0.20 (Gignac and Szodorai, 2016). Therefore, values of <0.10 can be taken to indicate the presence of *relatively small* magnitude effects (Gignac and Szodorai, 2016).

The effect is not confounded with (biogeographic) regional influences, age (which is a negative predictor of participant EA), parental EA (which unsurprisingly is a relatively large magnitude positive predictor of offspring EA), or social class (which is also an independent, albeit relatively small-magnitude, positive predictor of participant EA). The (relatively small magnitude) impact on participant EA of age indicates that older participants have lower EA, consistent with the expectation that younger participants are likely to have benefited from expansion of access to education through modernization (Barro and Lee, 2013). Reduced parent-offspring resemblance for EA among those with “higher” social class levels is not confounded with the various interactions among these main effects either. One notable interaction concerned the positive influence of participant age on social class as a predictor of participant EA. This indicates that while social class is a stronger predictor of EA among older participants, there appears to be increased social mobility with respect to educational opportunities among younger participants.

The quantile regression also found that among those with lower social class ordinals, the strength of the association between parental and participant EA was stronger relative to those with the highest social class ordinal. This result indicates that the finding of an effect consistent with the CAH in these data is robust to the use of different measurement models.

This apparent support for the CAH using a globally representative individual-differences dataset suggests that at the macrosocial scale, access to EA-enhancing resources associated with “higher” social class has (at least historically) had the effect of positively amplifying EA across generations in such a way that reduces parent-offspring resemblance for this outcome.

The precise mechanism through which the CAH may be operating in these data is unclear, however, as parent-offspring resemblance cannot distinguish between environmental and genetic influences on trait development (Devlin et al., 1997). Ruks’ (2022) behavior-genetic study of the CAH in a German twin sample found that the moderating effect of socioeconomic status on the heritability of an EA measure (specifically tertiary enrollment) is mediated by genetic variance associated with cognitive ability. This could explain results of the current study that are consistent with the action of gene-by-environment interactions.

It is important to note that gene-by-environment interactions are not the only pathway through which the CAH might operate. Interactions between and among bioecological factors (such as social class) and behavior-genetic variance components, capturing different aspects of environmentality, might also reduce parent-offspring resemblance for EA. Significant *environment-by-environment interactions* (Hanscombe et al., 2012) on cognitive ability and related phenotypes are seldom reported in the literature, however Hur and Bates (2019) found that despite the absence of a Scarr-Rowe effect in their study of Nigerian twins, there was nevertheless a significant interaction between a measure of familial chaos and shared environmentality (C) predicting cognitive ability. A small number of prior studies have also noted similar effects. In a large behavior-genetic study from the UK involving twins aged from 2 to 14 years, Hanscombe et al. (2012) noted an apparent C-by-SES interaction, such that among those with lower SES, C variance was greater for cognitive ability than among those with higher SES, even though, as with Hur and Bates (2019), no Scarr-Rowe effect was found. In a study involving German child twins, a significant interaction between non-shared environmentality (E) and SES (proxied by parental education) was noted predicting participant verbal performance (Spengler et al., 2018). The meta-analysis of Tucker-Drob and Bates (2015), on the other hand, found no evidence for these environment-by-environment interaction effects, when these were estimated with respect to both environmental variance components for the full set of studies.

A point needs to be made concerning the relatively small magnitude of the interaction terms identified in the current study, which has been used by some to dismiss the theoretical significance of prospective gene-by-environment and related effects more generally (for discussion see McGue and Carey, 2017). This situation is, however, consonant with the expectation that, when present, such interactions *should* be smaller than their associated main effects as the interactions will likely only influence trait variance to a small degree when considered independently, but may have additively larger impacts when grouped (McGue and Carey, 2017). As the CAH effect was estimated with respect to a *single* bioecological factor (social class) in the current study, its relatively low magnitude is therefore in line with theoretical expectations. It is furthermore increasingly clear that the nature of these interactions, both in relation to cognitive ability and related traits or outcomes such as EA, is much more complex than Sandra Scarr and other researchers initially thought. Theoretical work such as that of Shanahan and Boardman (2009) helps in making sense of the broader pattern of findings that have now emerged in the half-century since the publication of Scarr-Salapatek (1971). Nonetheless, it is apparent that bolder general theorizing concerning these interactions, and predictions about where and when they will be present (in either direction) or absent altogether are needed in order for scientific progress to continue in this area.

Given this need, presented here is some speculative theorizing about the nature of the effects under consideration as well as some testable predictions that emerge from this. Scarr-Rowe and CAH research might best advance if it is integrated within a broader behavioral and evolutionary ecology framework describing how and why organisms vary along a preparedness-plasticity axis. Preparedness is the “degree to which an organism is genetically predisposed toward a particular developmental trajectory, whereas [plasticity] constitutes the degree to which gene–environment interaction induced phenotypic changes during development may alter that prepared trajectory” (Woodley of Menie et al., 2015, p. 2). Properly understanding the Scarr-Rowe and CAH effects likely requires that the environmental factors modulating the degree of plasticity-preparedness a person will exhibit in development are taken into account.

A reasonable prediction is that human populations contending, to a rather uniform degree, with especially harsh environments—due, for example, to very low GDP *per capita*—will be epigenetically biased in development to exhibit lower plasticity and higher preparedness, as reduced sensitivity to environmental insults in ontogenetic time reduces the potential fitness costs of such insults. Societies with very and pervasively low absolute material quality of life might then fail to exhibit either Scarr-Rowe or CAH effects, due to unfavorable environments generally constraining realization of developmental plasticity (such environments likely also constrain the development of many traits to the full genetic potential of individuals who are raised therein). Conversely, societies high in inequality of material quality of life, but whose poor have benefited substantially from modernization, may exhibit both the Scarr-Rowe and CAH effect simultaneously, with the highest heritability of traits and outcomes such as cognitive ability and EA observed in those with mid-level SES. This is because in such societies, environments may be good enough for even those quite low in SES to be epigenetically predisposed to manifest developmental plasticity to such a degree that the heritability of certain traits is reduced for them relative to other SES groups within their society, via gene-by-environment interactions associated with the harmful influences of low-SES environments, giving rise to the Scarr-Rowe effect; and concomitantly, those with very high SES will likely tend to exhibit higher plasticity due to their favorable environments, which would be leveraged to compensate for diathesis at the level of genetic potential, again through gene-by-environment interactions, giving rise to the CAH effect. A society that prospectively meets these criteria, and would be predicted to exhibit both Scarr-Rowe and CAH effects, would be that of India, owing to the presence of an increasingly Westernized middle class, coupled with extremes of both poverty and wealth. Very large samples would be needed to properly saturate models, however (as a large number of non-linear, in addition to linear interactions would need to be estimated in addition to the effect of interest).

## Data availability statement

Publicly available datasets were analyzed in this study. This data can be found here: https://www.worldvaluessurvey.org/WVSNewsShow.jsp?ID=428.

## Ethics statement

Ethical review and approval was not required for the study on human participants in accordance with the local legislation and institutional requirements. Written informed consent from the patients/participants or patients/participants legal guardian/next of kin was not required to participate in this study in accordance with the national legislation and the institutional requirements.

## Author contributions

MW: Conceptualization, Writing – original draft. MS: Writing – original draft. MP-A: Writing – original draft. HR: Writing – review & editing.

## Footnotes

^1^For more information, see https://www.worldvaluessurvey.org/WVSContents.jsp.
